# A Glucagonoma Presenting as Cerebral Vein Thrombosis and Diabetes

**DOI:** 10.1155/2022/7659341

**Published:** 2022-04-22

**Authors:** Marina Delli Colli, Bader N. Alamri, Laura Palma, Juan Rivera

**Affiliations:** ^1^Division of Endocrinology and Metabolism, Department of Medicine, McGill University, Montreal, Quebec, Canada; ^2^Division of Medical Genetics, Department of Specialized Medicine, McGill University, Montreal, Quebec, Canada

## Abstract

Glucagonomas are rare pancreatic neuroendocrine tumors (pNETs), malignant in 80% of cases, thus highlighting the importance of early diagnosis and treatment. Primary manifestations are diabetes, dermatosis, depression, weight loss, and deep vein thrombosis. Unlike other pNETs, glucagonomas are associated with a higher incidence of thromboembolic events, often resulting in death. We present the case of a glucagonoma patient whose primary manifestation was cerebral sinus venous thrombosis (CS-VT). Early diagnosis enabled curative resection. The purpose of this paper is to review the underlying mechanisms associated with increased coagulopathy in glucagonomas.

## 1. Background

Glucagonoma is a rare, functional, pancreatic neuroendocrine tumor (pNET) in which the islet alpha cells of the pancreas oversecrete glucagon [1]. Accounting for only 4% of all pNETs, glucagonomas have a malignancy rate of 80% and therefore require early detection and treatment [2]. Their typical presentation, glucagonoma syndrome, is a paraneoplastic syndrome characterized by diabetes, dermatosis, and weight loss. Additionally, patients often present with anemia, malnutrition, diarrhea, glossitis, cheilitis, depression, and other psychiatric manifestations, and deep vein thrombosis [3, 4]. Herein, we present the case of a patient with a glucagonoma whose primary manifestation was cerebral sinus venous thrombosis (CS-VT), which to our knowledge has not been reported before.

## 2. Case Report

A 68-year-old man with a history of psoriasis and benign prostate hyperplasia experienced severe headaches, insomnia, and dizziness for three consecutive days. On the fourth day post symptom onset, he presented to the emergency department due to a worsening right-sided headache, along with new-onset vomiting and left-sided weakness/discoordination. A brain CT scan showed multicompartmental right parietal hemorrhage adjacent to the superior sagittal sinus with a surrounding meningioma. A CTA scan revealed an extensive superior sagittal sinus thrombosis extending into the right transverse and right sigmoid sinus. Treatment with intravenous heparin was initiated. During his hospitalization, the patient's headache improved, and his left-sided weakness resolved within one week. Left sensory ataxia remained. Workup for occult malignancy revealed, on a CT abdomen, an exophytic hyperdense enhancing lesion, measuring 1.1 × 2.2 cm, in the posterior aspect of the tail of the pancreas, suggestive of a neuroendocrine tumor. There was no evidence of primary malignancy or metastatic disease involving the chest, liver, or prostate. During this admission, a new diagnosis of hypertension and type 2 DM were made as he remained hyperglycemic, and his HbA1C was 11.8%. The patient denied a history of diarrhea, flushing, or abdominal pain but endorsed night sweats and weight loss. His family history was significant for a brother with a pancreatic neuroendocrine tumor (pNET) and sister with multiple meningiomas. Endocrinology was consulted, and a pertinent workup for pNET was ordered. Chromogranin A (CgA) was elevated at 439.9 ng/ml (≤82); however, the patient was already on proton pump inhibitors (PPIs). The urinary cortisol was normal. PTH, serum calcium, and the pituitary hormonal panel were all normal. hCG and CEA were also normal. Factor V Leiden and prothrombin were normal. Given the combination of new-onset DM, DVT, and pNET, serum glucagon was requested and reported as increased at 202 pg/ml and 174 pg/ml (reference range <80 pg/ml). A pancreatic MRI identified a well-defined round, T2 iso/hypointense lesion, measuring 10 x 11 × 10 mm in keeping with a pNET. A Ga68-DOTATATE PET scan identified the pancreatic lesion as DOTATATE-avid. Additional lesions were identified in the cranial cavity, compatible with meningiomas.

Based on the unusual presentation of pNET in two siblings, the patient was assessed in medical genetics and underwent germline testing for an 86-gene panel through a commercial laboratory (Invitae). No pathogenic/likely pathogenic variant was identified by next-generation sequencing, including all possible candidate genes known to be associated with hereditary predisposition to neuroendocrine tumors (i.e., *MEN1*, *VHL*, *NF1, NF2, TSC1, TSC2, PRKAR1A, PTEN, TP53, SMARCB1,* and *SMARCE1*). Two heterozygous variants of uncertain significance not thought to be associated with the patient's phenotype were detected, namely, *TSC2* (c.1952C > T) and *NBN* (c.806G > A). To date, the patient's brother with pNET has not been assessed in genetics.

The patient underwent laparoscopic distal pancreatectomy and splenectomy, with no complications. Pathology described a well-demarcated, white appearing, firm, homogenous nodule, measuring 13 x 10 × 9 mm, pT1N0M0, with a Ki-67 index of 2% (G1). The tumor cells showed moderate and diffuse glucagon staining with patchy insulin staining. Plasma glucagon decreased from 202 pg/ml to 47 pg/ml post surgical resection. Although measured while on an anti-factor *X* inhibitor (DOACs), LMWH anti-factor *X* was low at 0.37 U/mL (0.50–1.00 U/mL) presurgical resection, and factor *X* level was normal at 0.78 U/mL (*N* = 0.50–1.50 U/mL) post surgical resection.

## 3. Discussion

Glucagonomas are estimated to have an incidence rate of 1 case in 20 million people per year [5, 6]. Cases are reported either equally in both sexes, or in some series, with a male predominance of 70% [7]. While most cases are sporadic, 3% of glucagonomas are associated with genetic disorders such as MEN 1 or von Hippel–Lindau syndrome, the latter being less prevalent [8]. Most patients present in the fifth to sixth decade of life, and over 80% have metastatic disease at the time of diagnosis, often invading lymph nodes, liver, and bones. They tend to be large at the time of diagnosis (2–30 cm), with circulating glucagon levels from 120 to over 20,000 pg/mL [7, 9]. The classical manifestations of the glucagonoma syndrome include diabetes or prediabetes, dermatosis (necrolytic migratory erythema), diarrhea, dementia-like manifestations, deep vein thrombosis, anemia, and weight loss (often cachexia). The case discussed here was unusual in that it was diagnosed early while measuring under 2 cm, it had no metastases, and only some of the classical clinical manifestations.

Although glucagonomas are associated with a high incidence of thromboembolic events and cerebral sinus venous thrombosis (CS-VT) as the presenting manifestations, as seen in our case, are novel and merit recognition. Thromboembolic events have been reported in 30–80% of glucagonoma patients, particularly DVT of lower limbs and pulmonary embolism (PE). Over 50% of deaths in patients with glucagonoma are directly attributed to thromboembolic events [10]. However, glucagonomas do not seem to be typically associated with other signs of disseminated coagulopathy, and glucagon is not known to influence the coagulation cascade [11]. Therefore, the mechanism for this coagulopathy, aside from prothrombotic factors which are common to all malignancies is unclear [12]. Increased production of factor *X* by the tumor alpha cells of the pancreas has been a suggested mechanism [10]. It is more likely that the prothrombotic state associated with glucagonomas is multifactorial which contributing factors include zinc depletion [13], a potential direct effect of glucagon on fibrinolysis [14], imbalanced reduction of prothrombotic and fibrinolytic factors associated with impaired liver protein synthesis capacity, hyperhomocysteinemia, and endothelial dysfunction from B12 deficiency related to malabsorption and malnutrition [15].

In 70 to 90% of patients, CS-VT presents as acute or subacute headache lasting 1–3 days. Focal neurological deficits (mono- or paraparesis or hemiparesis) occur in about half of patients, hours or days after headache onset [16]. Glucagonomas, on the other hand, are also associated with neurological disturbances, occurring in 20% of patients [17]. These manifestations are highly diverse, including muscle weakness, hyperreflexia, insomnia, psychosis, depression, and overt dementia [17]. Our patient reported dizziness and insomnia prior to his admission to the hospital. Left hemiparesis was also present.

While literature suggests varying severities of glucose intolerance associated with glucagonoma, DM remains a cardinal sign of glucagonoma [17]. Glucagon's role in glucose homeostasis is mediated by its catabolic effects from stimulations of gluconeogenesis, glycogenolysis, ketogenesis, and lipolysis [10]. In addition to hepatic phosphorylase activation, glucagon stimulates insulin secretion and inhibits pancreatic enzymes excretion. The overall result is hyperglycemia [10]. Persistent hyperglucagonemia leads to the reduction of plasma amino acid concentrations, with amplification of essential amino acid catabolism [15]. Consequently, DM develops in 80% of glucagonoma patients along with muscle, fat mass, and glycogen store depletion [10, 15].

Weight loss is a very common finding in the initial presentation of glucagonomas, occurring in 56% to 91% of cases [17]. The catabolic action of glucagon directly causes nutritional and metabolic deficiencies of zinc, essential fatty acids, and amino acids [18]. Subsequent weight loss occurs as the hormone diverts dietary amino acids into glucose by the liver and away from protein synthesis by other tissues [11]. In link, glucagon induction of inflammatory mediators, such as arachidonic acid derivatives, contributes to generalized malabsorption and malnutrition [18]. Malnutrition is also worsened due to diarrhea, another common clinical feature of glucagonoma [19]. Diarrhea not only results from the increased glucagon levels but also from the cosecretion of gastrin, VIP, serotonin, or calcitonin [5]. This patient did not experience diarrhea, however, validated significant weight loss.

Such nutritional deficiency influences the integumentary system, causing cutaneous lesions characteristic of the glucagonoma syndrome [10]. Occurring in 70% of patients, necrolytic migratory erythema (NME) consists of erythematous scaling and crusting, often appearing in the groin and perineal areas [10]. Hypoaminoacidemia, vitamins B, and zinc deficiency are recognized causes [10]. As NME is most likely a late manifestation of years of tumor growth, early recognition of these lesions is crucial in preventing further risk of metastases [10]. Our patient's small tumor, with only mild elevation of plasma glucagon, was not associated with NME.

In summary, the case described here is a reminder to clinicians to keep alert for clues pointing in the direction of a treatable common denominator in a patient with an atypical constellation of symptoms or atypical presentation of common conditions. In reference to glucagonomas, we would like to highlight the fact that staying alert to concurrent clinical manifestation in the case of PNETs may lead to early recognition of this deadly tumor. At such early stage of the evaluation, surgical resection can potentially lead to a cure.

## Data Availability

All the data used to support the findings of this study are included in the article.
